# Integrated analysis of miRNA and mRNA paired expression profiling of prenatal skeletal muscle development in three genotype pigs

**DOI:** 10.1038/srep15544

**Published:** 2015-10-26

**Authors:** Zhonglin Tang, Yalan Yang, Zishuai Wang, Shuanping Zhao, Yulian Mu, Kui Li

**Affiliations:** 1 The State Key Laboratory for Animal Nutrition, Institute of Animal Science, Chinese Academy of Agricultural Sciences, Beijing 100193, China; 2 Agricultural Genome Institute at Shenzhen, Chinese Academy of Agricultural Sciences, Shenzhen, 518124, China; 3 Institute of Animal Science, Anhui Academy of Agricultural Sciences, Hefei, 230031, P. R. China

## Abstract

MicroRNAs (miRNAs) play a vital role in muscle development by binding to messenger RNAs (mRNAs). Based on prenatal skeletal muscle at 33, 65 and 90 days post-coitus (dpc) from Landrace, Tongcheng and Wuzhishan pigs, we carried out integrated analysis of miRNA and mRNA expression profiling. We identified 33, 18 and 67 differentially expressed miRNAs and 290, 91 and 502 mRNA targets in Landrace, Tongcheng and Wuzhishan pigs, respectively. Subsequently, 12 mRNAs and 3 miRNAs differentially expressed were validated using quantitative real-time PCR (qPCR), and 5 predicted miRNA targets were confirmed via dual luciferase reporter or western blot assays. We identified a set of miRNAs and mRNA genes differentially expressed in muscle development. Gene ontology (GO) enrichment analysis suggests that the miRNA targets are primarily involved in muscle contraction, muscle development and negative regulation of cell proliferation. Our data indicated that more mRNAs are regulated by miRNAs at earlier stages than at later stages of muscle development. Landrace and Tongcheng pigs also had longer phases of myoblast proliferation than Wuzhishan pigs. This study will be helpful to further explore miRNA-mRNA interactions in myogenesis and aid to uncover the molecular mechanisms of muscle development and phenotype variance in pigs.

MicroRNAs (miRNAs) are evolutionary conserved, small (~22 nt) non-coding RNAs that are found in all metazoans and regulate diverse biological processes^1^,^2^,^3^,^4^,^5^. miRNAs regulate gene expression by base pairing with their target mRNAs, leading to mRNA cleavage or translational repression^5^,^6^. Hence, the identification of the protein-coding targets of miRNAs is crucial for understanding their biological function. Several computational methods (PicTar, Target-Scan and miRanda) have been developed for the prediction of miRNA targets^6^,^7^,^8^,^9^. Although these programs are valuable for guiding laboratory experiments, they lack sensitivity and specificity^10^,^11^. A clear correlation is known to exist between the expression patterns of miRNAs and their mRNA targets^11^,^12^,^13^, and miRNA-target relationship analysis has been increasingly used to identify potential interactions between microRNAs and mRNAs based on paired expression profiles^14^,^15^,^16^,^17^,^18^,^19^.

During muscle development, miRNAs play crucial regulatory roles^20^,^21^,^22^,^23^,^24^,^25^,^26^,^27^,^28^,^29^. For example, miR-1, miR-133 and miR-206 are specifically and abundantly expressed in muscle tissue and contribute to muscle development^21^,^30^,^31^,^32^,^33^,^34^,^35^. miR-1 and miR-133 are involved in myoblast proliferation and differentiation^21^,^36^,^37^, and miR-206 has also been shown to promote myoblast differentiation^30^,^38^. Our group reported that miR-148a could promote myogenic differentiation by repressing the *ROCK1*^39^. The miRNAs represent a newly recognized level of regulation of gene expression mediating skeletal muscle development. However, the function of miRNAs in skeletal muscle development and phenotypic variance has remained largely unclear.

Prenatal skeletal muscle development (myogenesis) is an ideal paradigm for understanding the molecular basis of cell lineage establishment and differentiation into specialized structures^40^,^41^. Furthermore, prenatal skeletal muscle development influences postnatal muscle performance^42^,^43^. Accordingly, myogenesis has become an extensively studied process in animal models^44^. In the pig (*Sus scrofa*), two waves of muscle fiber formation occur over relatively long periods of time compared to other laboratory animals, making the pig a good animal model for studying myogenesis^45^. Postnatal muscle growth is determined by the total number of fibers (TNF), which is fixed before birth. Establishment of the TNF involves two major waves of fiber generation: a primary generation from 35 to approximately 60 days post coitus (dpc) and a secondary generation from approximately 54 to 90 dpc^43^. Hence, approximately 35, 60 and 90 dpc are considered the key time points in myogenesis that significantly contribute to postnatal phenotype variance in pigs^46^.

The pig is an important livestock animal and an ideal model for biomedical research due to its anatomical, physiological and genetic similarities to humans^47^,^48^,^49^,^50^,^51^,^52^. In addition, studies on pigs have aided our understanding of phenotypic variation and abundant phenotypic changes during long-term selection^53^. Therefore, the use of pigs as research animals benefits both biomedical research and animal agriculture^46^. Between different types of pigs (lean-, obese- and mini-types), there are significant differences in growth rate, muscle mass, meat quality and adult weight. Landrace pigs, which are a typical lean-type western breed, have been intensively selected over the past three decades for rapid, large and efficient accretion of muscle. Tongcheng pigs, which are a typical indigenous Chinese obese-type breed, have a markedly lower growth rate and higher fat content than lean-type western pig breeds. Wuzhishan pigs, which are a Chinese miniature breed, have been recognized as an attractive experimental animal for a wide range of research fields (adults weigh < 40 kg)^54^,^55^.

To explore molecular mechanism of prenatal muscle development in pigs, we integrated miRNA- and mRNA-paired expression profiling of prenatal skeletal muscle at 33, 65 and 90 dpc in Landrace (lean-type), Tongcheng (obese-type) and Wuzhishan (mini-type) pigs. We identified miRNAs and mRNAs that were differentially expressed during muscle development. Subsequently, we performed expression pattern and co-expression analysis of differentially expressed mRNA during prenatal skeletal muscle development in three breed pigs. And a mRNA-miRNA interaction study was also carried out using computational prediction and expression relationship analysis. Finally, 12 mRNAs and 3 miRNAs that were differentially expressed were validated by quantitative real-time PCR (qPCR), and 5 predicted miRNA targets were validated via dual luciferase reporter or western blot assays.

## Results

### Identifying differentially expressed mRNAs and miRNAs

During prenatal skeletal muscle development, 1402, 912 and 1950 transcripts were differentially expressed (*P* < 0.05) in Landrace, Tongcheng and Wuzhishan pigs, respectively (Tables S1–3). We also identified 33, 18 and 67 differentially expressed miRNAs (*P* < 0.05) in Landrace, Tongcheng and Wuzhishan pigs, respectively (Tables S4–6). Most miRNAs were differentially expressed in one pig breed, but we found that nine miRNAs (miR-133b, miR-206, miR-202, miR-302b, miR-320, miR-500, miR-625, miR-665 and miR-700) were common to all three breeds. However, there were varied expression patterns for the muscle-specific miRNAs miR-133b and miR-206 in each breed. miR-133b was up-regulated in both Landrace and Tongcheng pigs, but down-regulated in Wuzhishan pigs. In Landrace pigs and Tongcheng pigs, miR-206 peaked at 90 dpc, but it peaked at 65 dpc in Wuzhishan pigs.

In addition, some miRNAs were differentially expressed in only one breed during myogenesis. For example, only miR-619 was differentially expressed in Landrace pigs, while miR-1 and miR-765 were differentially expressed in Tongcheng pigs. In Wuzhishan pigs, 32 miRNAs, including let-7b, let-7d and let-7i from the let-7 family, were differentially expressed during myogenesis. These miRNAs maybe significantly contribute to the characteristic muscle phenotype of each pig breed.

### qPCR validation for miRNAs and mRNAs

We selected three miRNAs (miR-133b, miR-206 and miR-302b) and twelve mRNAs (*IGF2, TPM3, COL15A1, CSRP3, LAMA2, LAMA3, LAMB2, UCHL1, FNDC1, FEZ2, FGL2* and *IGFBP7*) to validate our miRNA and mRNA microarray data by qPCR. As shown in Table 1, the qPCR results were in agreement with the miRNA microarray experiments (r = 0.7937, *P* < 0.001). miR-133b was up-regulated during myogenesis in both Landrace and Tongcheng pigs but down-regulated in Wuzhishan pigs. miR-206 was up-regulated in both Landrace and Tongcheng pigs and peaked at 65 dpc in Wuzhishan pigs. miR-302 was down-regulated in Landrace and Tongcheng pigs but up-regulated in Wuzhishan pigs. As shown in Table 2, both the qPCR and oligoarray data validated that *COL15A1, IGF2, CSRP3, CSRP3, LAMA3, LAMB2, FEZ2, FGL2* and *IGFBP7* were up-regulated, *UCHL1* was down-regulated, and *FNDC1* peaked at 65 dpc in the three pig breeds; in addition, *TMP3* was up-regulated in both Landrace and Wuzhishan pigs and peaked at 65 dpc in Tongcheng pigs. The relationship coefficient between the oligoarray and qPCR data was 0.7511 (*P* < 0.01). These findings indicated that our miRNA and mRNA microarray data reliably reveal differences in the myogenesis gene expression profiles of these three breeds.

### Co-expression analysis of mRNAs during skeletal muscle development

Genes with parallel expression pattern are usually involved in the similar biological functions and pathways^56^,^57^. To identify key regulatory genes and compare the differences in mRNA expression during muscle development of different breeds, we performed gene expression pattern analysis using STEM software. These expression patterns are shown graphically for each breed in Figure S1–3 for Landrace, Tongcheng and Wuzhishan pigs, respectively. The results showed that differentially expressed genes were significantly enriched in 7, 7 and 6 clusters for Landrace, Tongcheng and Wuzhishan pigs, respectively (*P* < 0.05).

Certain GO functional categories of genes were over-represented in a number of expression pattern clusters (Table 3). In Landrace, genes with up-regulated patterns in development were significantly enriched in cluster 15, 12, 11 and 13, the genes of each cluster were significantly involved in myofibril assembly, striated muscle contraction, muscle system process and vasculature development, respectively. Down-regulated genes were significantly enriched in cluster 0, 3 and 2, the genes of each cluster were mainly associated with DNA metabolic process, cell division and cytoskeleton organization, respectively. In Tongcheng pigs, genes with up-regulated patterns in development were significantly enriched in cluster 15, 13, 12 and 8. Of them, genes were significantly involved in striated muscle contraction, respiratory electron transport chain, negative regulation of biological process and cellular respiration, respectively. The down-regulated genes were significantly enriched in cluster 3, 7 and 2, we found that genes involved in cell division, actin-mediated cell contraction and protein polymerization were significantly enriched in these clusters. Comparing with Landrace and Tongcheng pigs, interestingly, Wuzhishan pigs exhibited significant differences in enriched expression patterns and GO categories of clusters. During skeletal muscle development, up-regulated genes were mainly enriched in cluster 11, 15 and 12 and significantly involved in signal transduction, energy reserve metabolic and somite development, respectively. Many genes exhibited a waved expression pattern (up-regulated from 33 to 65 dpc, than down-regulted from 65 to 90 dpc), these genes were mainly involved in single-organism organelle organization and enriched in cluster 14. We found that down-regulated genes were significantly enriched in cluster 4 and 1. Of them, genes were mainly associated with glucose metabolic process and generation of precursor metabolites and energy.

**Figure 1:**
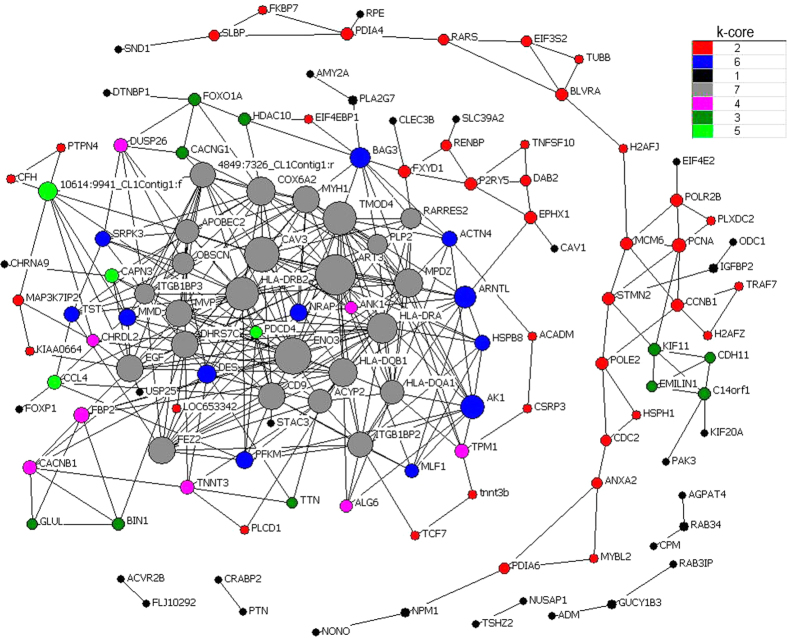
Gene co-expression network in Landrace pigs. Genes from cluster 15 and 0 were analyzed and identified by gene co-expression network with k-core algorithm. Cycle nodes represent genes, the size of nodes represents the power of the interrelation among the nodes, and edges between two nodes represent interactions between genes. The higher k-core of a gene means the more central role it has within the network.

**Figure 2:**
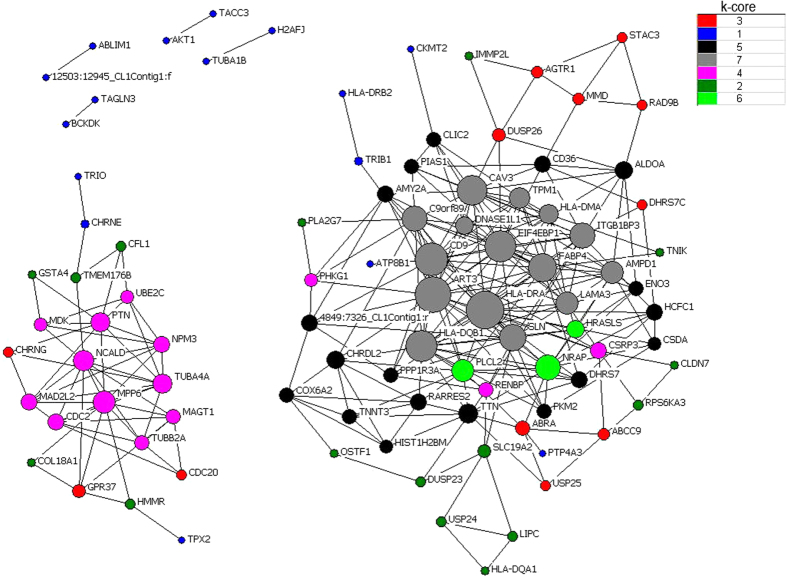
Gene co-expression network in Tongcheng pigs. Genes from cluster 15 and 3 were analyzed and identified by gene co-expression network with k-core algorithm. Cycle nodes represent genes, the size of nodes represents the power of the interrelation among the nodes, and edges between two nodes represent interactions between genes. The higher k-core of a gene means the more central role it has within the network.

**Figure 3:**
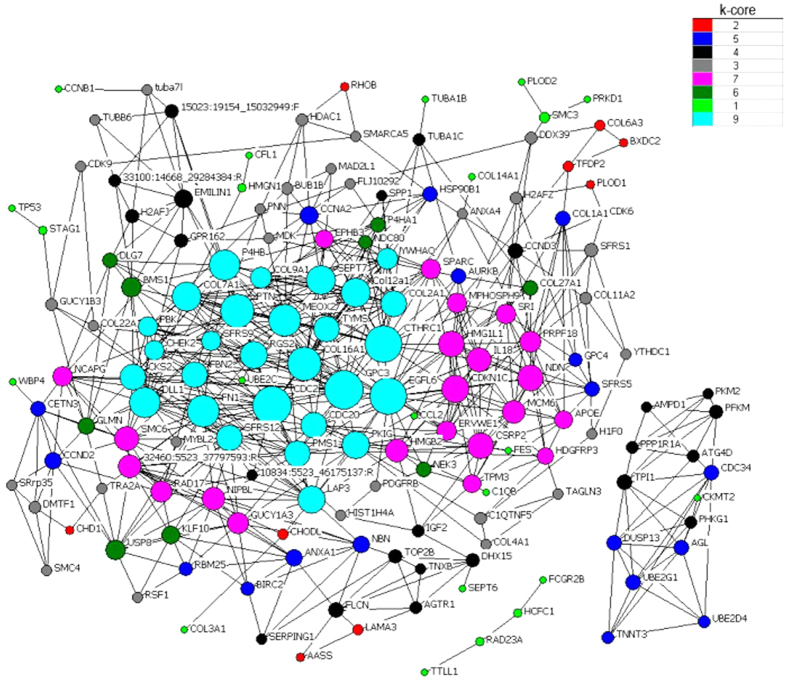
Gene co-expression network in Wuzhishan pigs. Genes from cluster 11 and 4 were analyzed and identified by gene co-expression network with k-core algorithm. Cycle nodes represent genes, the size of nodes represents the power of the interrelation among the nodes, and edges between two nodes represent interactions between genes. The higher k-core of a gene means the more central role it has within the network.

Subsequently, we focused on the top significantly clusters with up-regulated and down-regulated patterns. co-expression and interaction analysis were carried out based above genes. In Landrace pigs, we found some key regulators (k-core = 7) including muscle contraction genes (*FXYD1*, *DES*, *TNNT3*, *CACNB1*, *CACNG1*, *GAMT* and *TMOD4*) and myogenesis factor (*TNNT3b*, *CAPN3*, *CAV3* and *ITGB1BP2*) in clusters 15 and 0 (Fig. 1). In Tongcheng pigs, three myogenesis-related genes (*MAPK12*, *CAV3* and *TAGLN3*) were identified as key regulatory factors (k-core = 7) for prenatal skeletal muscle development in clusters 15 and 3 (Fig. 2). In Wuzhishan pigs, key regulators (k-core = 9) were significantly involved in the cell proliferation process (including *BUB1B*, *GPC4*, *LGI1*, *IGF2*, *TP53*, *FES*, *PTN*, *MDK*, *CKS2*, *USP8*, *CDK9*, *CDK6*, *KLF10*, *PRKD1*, *DLG7*, *CSRP2* and *HDGFRP3*) and myogenesis (including *TNXB*, *GDF*8, *TPM3*, *SRI*, *CSRP2*, *CHODL* and *TAGLN3*) in cluster 11 and 4 (Fig. 3).

### miRNA-mRNA interaction analysis

#### In Landrace pigs

**Figure 4:**
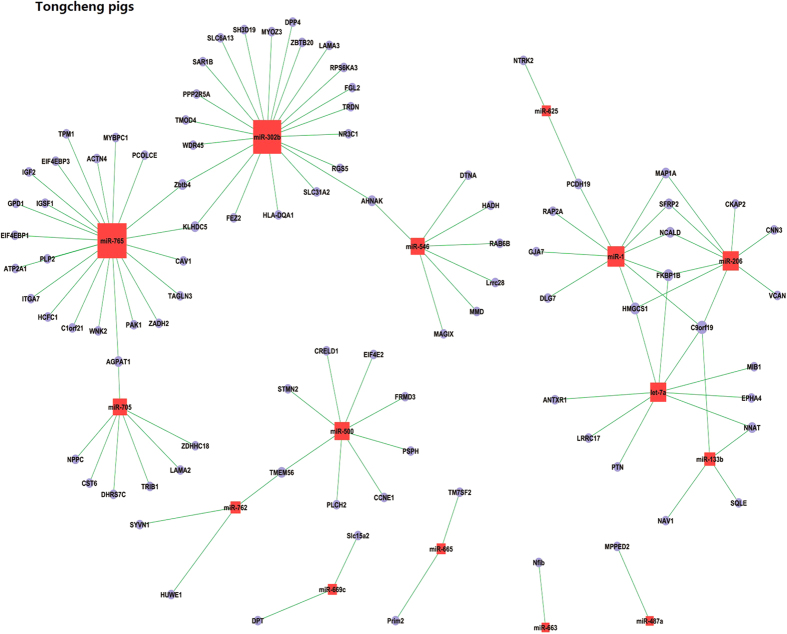
The miRNA-mRNA interaction based on correlation analysis of their expression during prenatal skeletal muscle development in Landrace pigs.

Using expression correlation and computational prediction, we identified 290 potential mRNA targets for 28 miRNAs based on paired miRNA and mRNA expression profiling (Table S4). The miRNA-mRNA interaction relationship for Landrace pigs is shown in Fig. 4. In Landrace pigs, 14 miRNAs (miR-608, miR-302b, miR-136, miR-625, miR-382, miR-207, miR-202, miR-619, let-7d, miR-376c, miR-665, miR-552, miR-697, miR-328 and miR-133b) were found to regulate 81.2% of the mRNA targets identified. Of these miRNAs, miR-608, miR-302b, miR-136 and miR-625 potentially targeted 52, 46, 36 and 36 mRNAs, respectively, indicating that these miRNAs likely contribute significantly to the regulation of mRNA expression during prenatal muscle development in Landrace pigs. miR-133b potentially targeted 12 mRNAs (*ABCF2*, *ARID3A*, *ATOX1*, *C9orf19*, *EIF4A1*, *HPGD*, *NNAT*, *ODC1*, *SCP2*, *SMARCD1*, *SMC2* and *SQLE*). *CKAP2*, *SFRS3*, *TPM3*, *YWHAB* and *YWHAQ* were identified as targets of miR-206. We found that one miRNA can regulate multiple target mRNAs; one mRNA can also be regulated by multiple miRNAs. For example, the expression of *CAV1*, an isoform of the muscle-specific caveolin gene family^58^, was regulated by miR-136, miR-302b and miR-552. In addition, *IGF2* (insulin-like growth factor 2), which is known to affect muscle growth in the pig^59^, was the target of both miR-552 and miR-625. Finally, expression of *NPM1*, which is involved in cell survival and proliferation during carcinogenesis^60^, was potentially modulated by miR-519d, miR-619 and miR-207.

#### In Tongcheng pigs

**Figure 5:**
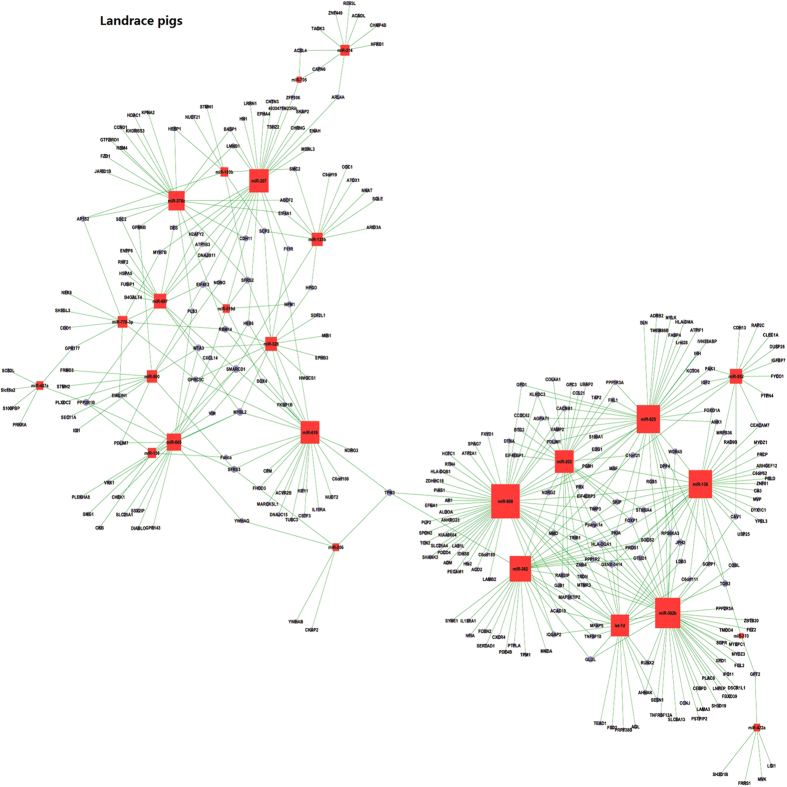
The miRNA-mRNA interaction based on correlation analysis of their expression during prenatal skeletal muscle development in Tongcheng pigs.

In Tongcheng pigs, we identified 91 potential mRNA targets for 15 miRNAs involved in myogenesis (Table S5). Eight miRNAs (miR-765, miR-302b, miR-1, let-7a, miR-206, miR-500, miR-546 and miR-705) targeted 86.1% of the mRNAs in Tongcheng pigs. There were 22, 21, 10, 9 and 9 target mRNAs for miR-765, miR-302b, miR-1, let-7a and miR-206, respectively, suggesting that these miRNAs significantly contribute to prenatal skeletal muscle development in Tongcheng pigs. The target mRNAs of miR-1, 133b and 206 were identified in this study. As shown in Fig. 5, miR-1 potentially regulated 10 mRNAs, including *C9orf19*, *DLG7*, *FKBP1B*, *GJA7*, *HMGCS1*, *MAP1A*, *NCALD*, *PCDH19*, *RAP2A* and *SFRP2*. miR-133b potentially regulated 4 mRNAs (*C9orf19*, *NAV1*, *NNAT* and *SQLE*), while miR-206 potentially regulated *C9orf19*, *CKAP2*, *CNN3*, *FKBP1B*, *HMGCS1*, *MAP1A*, *NCALD*, *SFRP2* and *VCAN*. The expression of *C9orf19* was potentially regulated by miR-1, miR-133b, miR-206 and let-7a. *FKBP1B* and *HMGCS1,* which encode rate-limiting enzymes of the cholesterol synthesis pathway^61^, were potentially regulated by let-7a, miR-1 and miR-206. The *SFRP*2 gene, which plays an active role in embryogenesis, especially in muscle development^62^, was found to be a potential target of miR-1 and miR-206 in myogenesis in Tongcheng pigs.

#### In Wuzhishan pigs

**Figure 6:**
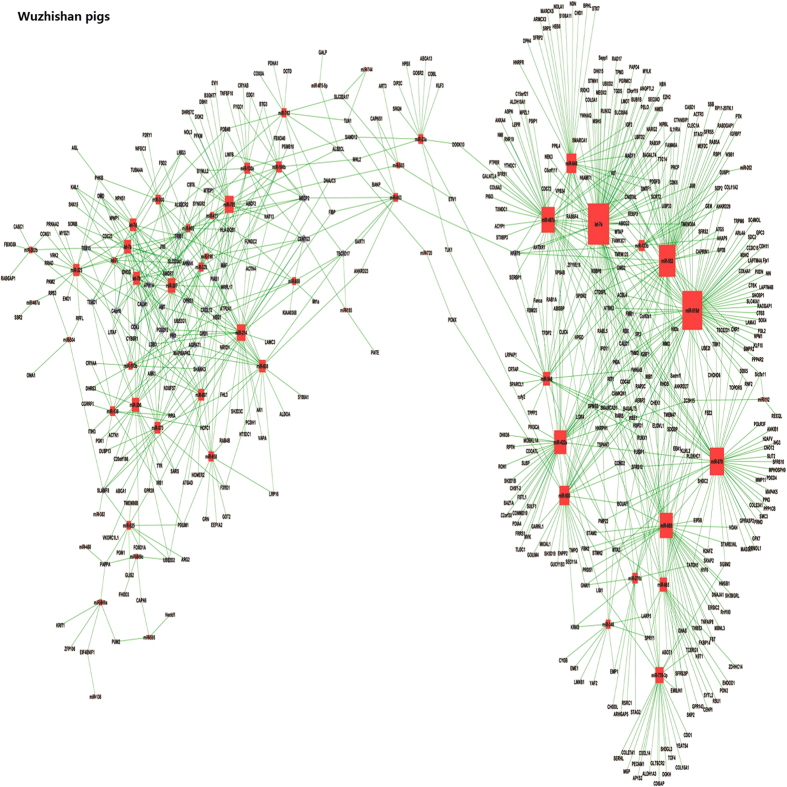
The miRNA-mRNA interaction based on correlation analysis of their expression during prenatal skeletal muscle development in Wuzhishan pigs.

By combining mRNA-miRNA paired expression and computational prediction, we identified 502 potential mRNA targets for 57 miRNAs associated with myogenesis in Wuzhishan pigs (Table S6). The prediction result of mRNA-miRNA interaction is shown in Fig. 6. In Wuzhishan pigs, 31 miRNAs potentially regulated 85.69% of mRNAs differently expressed during prenatal skeletal muscle development. Of these mRNAs, more than half (51.07%) were potentially regulated by 11 miRNAs (let-7e, miR-519d, miR-552, miR-679, miR-487a, miR-685, miR-422a, miR-500, miR-648, miR-705 and miR-214). One miRNA was found to regulate multiple mRNAs. For example, let-7e, miR-519d, miR-552, miR-679 and miR-487 potentially targeted 77, 72, 58, 49 and 45 mRNAs, respectively. For miRNA-133b, we identified 15 potential mRNA targets (*AKAP9*, *ANKRD29*, *CHCHD6*, *DUSP1*, *ELOVL1*, *FAM46A*, *GEM*, *GNG2*, *MACF1*, *NFAT5*, *PRCP*, *RAP2C*, *SCP2*, *SCRT2* and *SPON2*) in Wuzhishan pigs. Twenty-one mRNAs (*ACTN1*, *AGPAT1*, *AMT*, *ANK1*, *C20orf186*, *CALM1*, *CCNJ*, *CXCL12*, *DHRS3*, *FOXO1A*, *IRS1*, *JTB*, *LDB3*, *LITAF*, *PGK1*, *RPIA*, *SARS*, *SLAMF8*, *TEAD1*, *TTR* and *UBE2D2*) were potentially regulated by miR-206 in Wuzhishan pigs. The expression levels of *CCNJ*, *CENTG3*, *POU2F2*, *PRX* and *TRIB1* were potentially regulated by 9 different miRNAs. We also found that *CCNJ* had potentially common target loci: let-7a, let-7b, let-7d, miR-198, miR-206, miR-207, miR-325, miR-370 and miR-697. Similarly, the dystrophin (*DMD*) gene, involved in Duchenne muscular dystrophy^63^, was potentially regulated by let-7a, let-7b, let-7d, miR-207 and miR-325. Finally, *IGF*2, which is up-regulated in myogenesis, was a potential target of let-7e, miR-552 and miR-648 in Wuzhishan pigs.

### Validating mRNA targets for miRNAs

**Figure 7:**
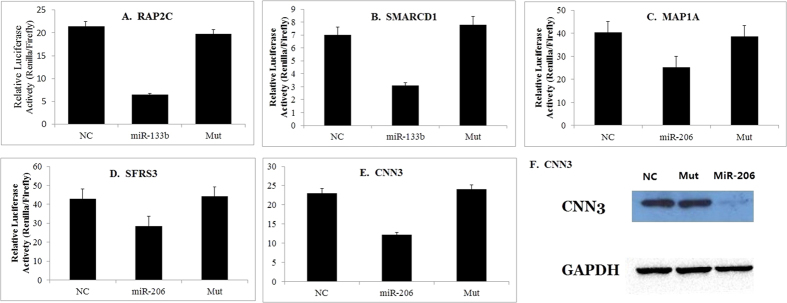
Validation of miRNA-mRNA interactions involving *RAP2C*, *SMARCD1*, *MAP1A*, *SFRS3* and *CNN3* via dual luciferase reporter or western blot assay. (**A**,**B**) Validating *RAP2C* and *SMARCD1* as targets of miR-133b using a dual luciferase reporter assay. (**C**–**E**) Validating *MAP1A*, *SFRS3* and *CNN3* as targets of miR-206 using a dual luciferase reporter assay. (**F**) Validating CNN3 as a target of miR-206 at the protein level using western blot.

According to the miRNA-mRNA interaction analysis and the prediction results of TargetScan and PicTar, *RAP2C* and *SMARCD1* were potential target genes of miR-133b, and *MAP1A*, *SFRS3* and *CNN3* were potential target genes of miR-206 (Table 4). To further assess the validity of miRNA-mRNA interactions, we chose these potential mRNA targets for experimental validation in pig iliac endothelium cell lines (PIEC). We carried out dual luciferase assays to confirm the binding potentiality between miRNAs and mRNA targets. The luciferase activity of wild-type of *RAP2C* and *SMARCD1* reporters co-transfected with miR-133b mimics was decreased 69.8% (*P* < 0.01) (Fig. 7A) and 56.3% (*P* < 0.01) (Fig. 7B) compared to that of the negative control, respectively. The luciferase activity of wild-type *MAP1A*, *SFRS3* and *CNN3* reporters co-transfected with miR-206 mimics was decreased 37.4% (*P* < 0.01) (Fig. 7C), 33.5% (*P* < 0.01) (Fig. 7D) and 47.3% (*P* < 0.01) (Fig. 7E), respectively, compared to the negative control mimics. Additionally, all of these repression were abrogated after mutating the putative binding sites of these targets genes (Fig. 7A–E). These results supported that ssc-miR-133b directly targeted the *RAP2C* and *SMARCD1* 3′UTR and ssc-miR-206b directly targeted the *MAP1A*, *SFRS3* and *CNN3* 3′UTR. Moreover, western blot suggested that miR-206 repressed the expression of CNN3 at the protein level (Fig. 7F).

### Gene ontology (GO) annotation of potential targets for miRNA

**Figure 8:**
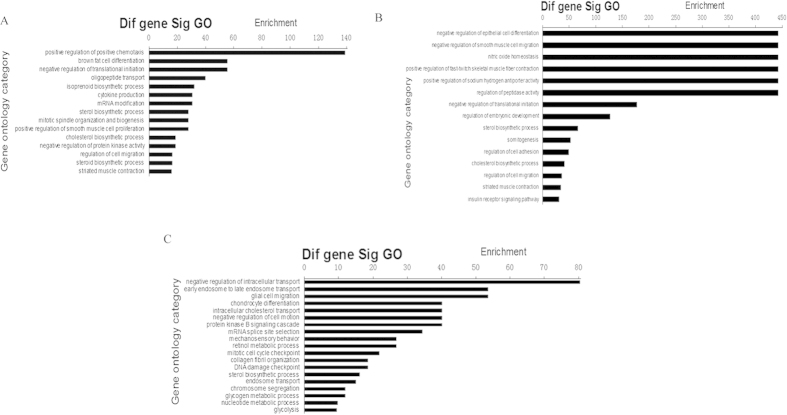
Significant GO categories of microRNA targets during prenatal skeletal muscle development of pigs. (**A**) Landrace pigs; (**B**) Tongcheng pigs; (**C**) Wuzhishan pigs. The vertical axis is the GO category, and the horizontal axis is GO enrichment.

**Figure 9:**
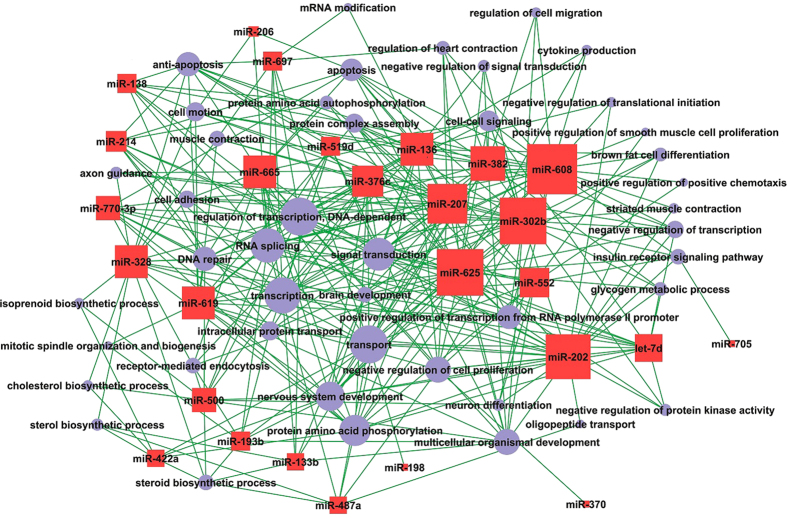
The miRNA-GO network during prenatal skeletal muscle development in Landrace pigs. Red squares indicate miRNAs that regulate myogenesis by targeting mRNAs. The pearl blue background indicates biological processes associated with mRNAs regulated by miRNAs in myogenesis.

To gain further insight into the biological processes potentially mediated by miRNAs in myogenesis, we analyzed the functional categories of target mRNAs by performing gene ontology (GO) analysis. In Landrace pigs, the high-enrichment GOs targeted by miRNAs included positive regulation of positive chemotaxis, negative regulation of translational initiation, and oligopeptide transport (Fig. 8A). The miRNA targets that negatively regulate cell proliferation were *CHEK1*, *TOB2*, *CDH13*, *CAV1*, *PRKRA*, *NPM1*, *IGFBP7*, *SESN1*, *GPC3*, *SKAP2* and *BTG2*, while the miRNA targets participating in muscle contraction were *TRDN*, *TMOD4*, *CHRNG*, *DES*, *FKBP1B* and *FXYD1*. As shown in Fig. 9, one miRNA could participate in several biological processes by targeting different mRNAs, and one biological process could be associated with multiple miRNAs. For example, miR-133b was associated with the sterol biosynthetic process, the steroid biosynthetic process, transcription, cell transport, regulation of transcription for DNA-dependent and multicellular organismal development. We found that negative regulation of cell proliferation was associated with several miRNAs, including let-7d, miR-136, miR-202, miR-302b, miR-370, miR-487a, miR-519d, miR-552, miR-619, miR-625, miR-207 and miR-665.

**Figure 10:**
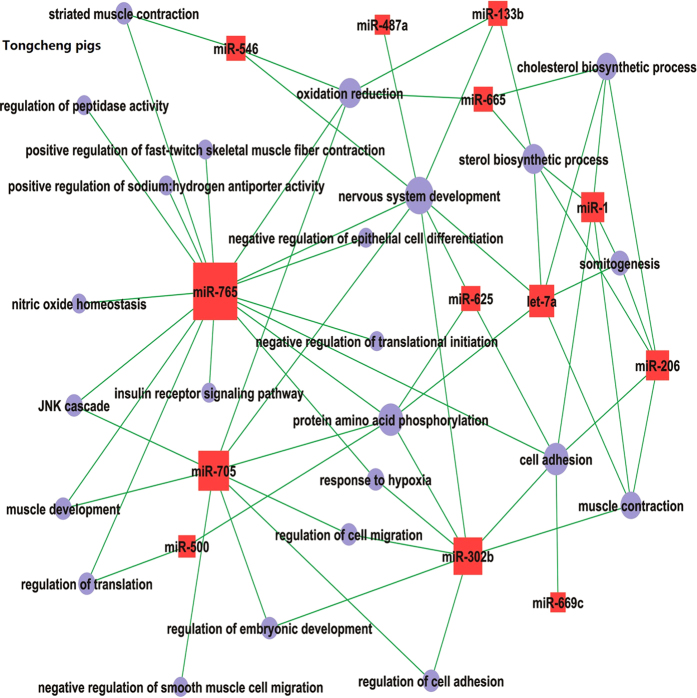
The miRNA-GO network during prenatal skeletal muscle development in Tongcheng pigs. Red squares indicate miRNAs that regulate myogenesis by targeting mRNAs. The pearl blue background indicates biological processes associated with mRNAs regulated by miRNAs in myogenesis.

In Tongcheng pigs, significant GOs corresponding to miRNAs included negative regulation of epithelial cell differentiation, negative regulation of smooth muscle cell migration, nitric oxide homeostasis and positive regulation of fast-twitch skeletal muscle fiber contraction (Fig. 8B). Interestingly, miR-765 participates in muscle development by targeting *ITGA7* and *TAGLN3*, which may be critical for differentiation and migration processes during myogenesis^64^. The miRNA-GO network analysis suggested that cell adhesion was regulated by six miRNAs (miR-1, miR-206, miR-302b, miR-625, miR-765 and miR-669c). miR-1 was associated with the sterol biosynthetic process, somitogenesis, muscle contraction, the cholesterol biosynthetic process and cell adhesion (Fig. 10).

**Figure 11:**
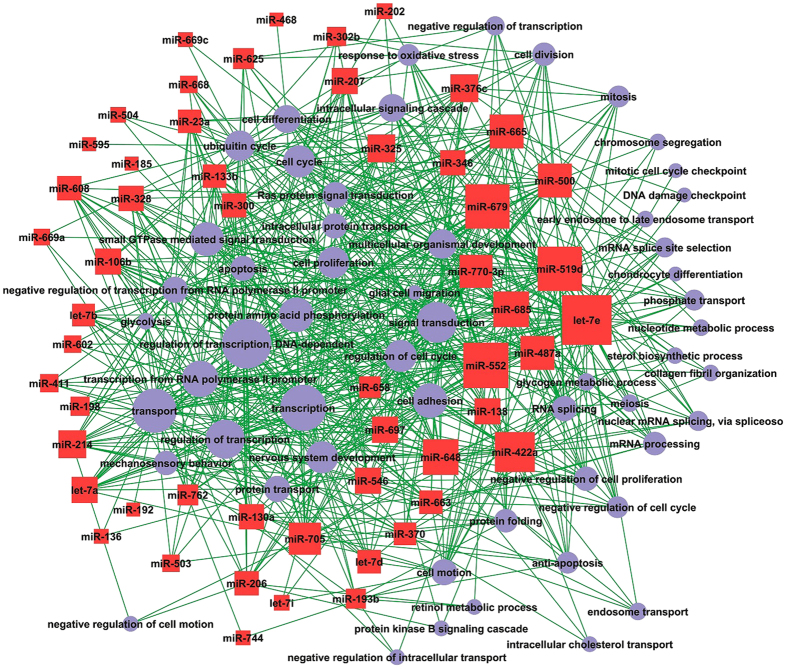
The miRNA-GO network during prenatal skeletal muscle development in Wuzhishan pigs. Red squares indicate miRNAs that regulate myogenesis by targeting mRNAs. The pearl blue background indicates biological processes associated with mRNAs regulated by miRNAs in myogenesis.

In Wuzhishan pigs, the significant GOs targeted by miRNAs in myogenesis included negative regulation of intracellular transport, early endosome to late endosome transport, glial cell migration and chondrocyte differentiation (Fig. 8C). We obtained the miRNA-GO network based on miRNA-mRNA interactions. As shown in Fig. 11, we found that cell division was associated with multiple miRNAs, including let-7e, miR-302b, miR-487a, miR-519d, miR-552, miR-207, miR-325, miR-346, miR-376c, miR-500, miR-679 and miR-770-3p. In addition, miR-206, miR-608, miR-325 and miR-762 were identified as regulators of glycolysis during prenatal skeletal muscle development. Finally, miR-133b was involved in the cell cycle, small GTPase-mediated signal transduction, cell adhesion, signal transduction, cell proliferation, the response to oxidative stress, regulation of transcription, DNA-dependent transcription from RNA polymerase II promoters and regulation of intracellular signaling cascades.

## Discussion

miRNAs regulate gene expression by binding to the 3′-untranslated region (UTR) of the target mRNA. It is estimated that each miRNA regulates hundreds of different mRNAs, and more than 60% of protein-coding genes are subject to miRNA regulation in the human genome. miRNAs directly regulate at least 30% of the genes in the human genome^7^,^65^. This fact suggests that miRNAs play an important role in gene expression for many biological processes. Thus, it is crucial to identify target mRNAs to understand the biological function of miRNAs. At present, however, it is difficult to identify miRNA-mRNA interactions^15^. In particular, the high false-positive rate (21–39%) of prediction programs is problematic, as it leads to laborious and time-consuming validation processes^7^,^10^,^66^. Additionally, computational prediction programs often return hundreds of mRNA targets for a given miRNA^65^. More recently, microarray-based techniques have been used to identify mRNA-miRNA interactions by determining negative expression correlations between miRNAs and their target mRNAs^11^,^14^,^15^,^16^,^17^,^18^,^67^,^68^. In this study, we carried out miRNA and mRNA paired expression profiling and then combined classical Pearson’s expression correlation analysis and computational programs to identify the miRNA-mRNA interactions potentiality during skeletal muscle development in three different breed pigs. This strategy significantly decreased the false-positive rate for identifying mRNA targets of miRNA. Using the Targetscan prediction program, for example, a total of 478, 435 and 478 mRNA targets were obtained for miR-1, miR-133 and miR-206, respectively. In this study, we identified only 10, 27 and 34 mRNA potential targets for miR-1, miR-133b and miR-206, respectively. A total of 732 potential mRNA targets were identified for 61 miRNAs expressed during prenatal skeletal muscle development in the pig. The number of target mRNAs ranged from 1 to 78 for a given miRNA, and one mRNA was potentially regulated by 1–12 miRNAs. Additionally, we validated interaction potentiality above prediction results based on five potential mRNA targets selected (*RAP2C* and *SMARCD1* for miR-133b, *CNN3*, *MAP1A* and *SFRS3* for miR-206) by experimental method. Our study revealed that the strategy combining expression profiling and computational prediction is an effective means for discovering miRNA-mRNA interactions. This study provides information that can be used to further characterize miRNA-mRNA interactions in myogenesis and understand phenotype variance of skeletal muscle in pigs.

Many studies have suggested that skeletal muscle development is regulated by miRNAs^21^,^24^,^28^,^29^,^39^,^69^,^70^. However, to date, only a few miRNAs involved in myogenesis have been identified. A previous study suggested that miR-1, miR-133b and miR-206 are associated with skeletal muscle development; we also found that these miRNAs were differentially expressed and critical to prenatal skeletal muscle development. It is currently estimated that miRNAs account for ~1% of predicted genes in eukaryotic genomes^71^, whereas more than 60% of human genes might be subject to regulation by miRNAs^65^. Most genes appear to be regulated by more than one miRNA. However, many miRNAs involved in muscle development remain unknown. Even in mammals, little is known about miRNA expression levels or patterns in myogenesis, and many miRNAs have yet to be discovered. We identified 75 differentially expressed miRNAs during prenatal skeletal muscle development in pigs. Of these miRNAs, nine (miR-133b, miR-206, miR-202, miR-302b, miR-320, miR-500, miR-625, miR-665 and miR-705) were differentially expressed in all three breeds. With the exception of miR-133b and miR-206, there have been no reports on the functions of these miRNAs in myogenesis. The present study discovered additional miRNAs involved in mammalian myogenesis.

Different breeds of pigs have significant postnatal phenotype differences in birth weight, growth rate and muscle mass, and these differences are programmed during prenatal muscle development. A previous study reported that approximately 35, 60 and 90 dpc were key time points in pig myogenesis^72^. To explore the contribution of miRNAs to muscle development and postnatal phenotype variance, we carried out paired miRNA and mRNA expression profiling of skeletal muscle at 33, 65 and 90 dpc in Landrace (lean-type), Tongcheng (obese-type) and Wuzhishan (mini-type) pigs. Our analysis suggests the existence of significant differences in the expression of miRNAs and mRNAs between breeds. There were differences in category of genes differentially expressed during myogenesis. Only nine miRNAs were expressed in all three breeds during myogenesis. Most miRNAs exhibited temporal and spatial specificity. In selected developmental muscle, while miR-1 and miR-765 were differentially expressed in Tongcheng pigs and 32 miRNAs were differently expressed uniquely in Wuzhishan pigs, only miR-619 was differentially expressed exclusively in Landrace pigs. Thus, these breed-specific miRNAs likely significantly contribute to the skeletal muscle development of each breed. Additionally, Tongcheng pigs are more similar to Landrace pigs than to Wuzhishan pigs in mRNA expression, especially with respect to skeletal muscle development at 33 and 65 dpc. Previous studies revealed that more miRNAs play regulatory roles in the embryonic mouse compare to postnatal animal. In this study, we also found that more miRNAs were differentially expressed at earlier stages (24 miRNAs at 33 dpc) than at later stages (12 miRNAs for 65 dpc and 12 for 90 dpc) between Landrace and Tongcheng pigs. Between Landrace and Wuzhishan pigs, more miRNAs were differently expressed at 33 dpc (54) and 65 dpc (51) than at 90 dpc (16). This trend held true for Tongcheng and Wuzhishan pigs (54, 47 and 18 miRNAs differentially expressed at 33, 65 and 90 dpc, respectively). These facts suggest that miRNAs play more important roles at earlier stages of myogenesis than at later stages of myogenesis.

For miRNA, it is critical to identify target gene for understanding its biological function and molecular mechanism. In this study, we identified 91 potential target mRNAs for 15 miRNAs in Tongcheng pigs, 290 potential target mRNAs for 28 miRNAs in Landrace pigs, and 502 potential target mRNAs for 58 miRNAs in Wuzhishan pigs during myogenesis. Unfortunately, we did not get mRNA target for some miRNAs, such as miR-106b and miRNA-125b in Landrace. The reasons maybe is that the prediction target genes were not differentially expressed at mRNA level in the dataset. For the nine miRNAs that were expressed in all three breeds, most target genes were unique to each breed, suggesting that most miRNAs regulate gene expression by targeting different mRNAs in the given tissues. GO enrichment analysis also suggested that different biological processes are potentially regulated by these miRNAs in the different types of pigs. In Tongcheng pigs (obese-type), the top ten biological processes mediated by these miRNAs were the sterol biosynthetic process, nervous system development, negative regulation of translational initiation, regulation of embryonic development, regulation of translation, protein amino acid phosphorylation, the response to hypoxia, somitogenesis, muscle development and regulation of cell adhesion. In Landrace pigs (lean-type), the top ten biological processes were negative regulation of cell proliferation, the sterol biosynthetic process, protein amino acid phosphorylation, RNA splicing, muscle contraction, positive regulation of positive chemotaxis, the isoprenoid biosynthetic process, DNA repair, cell-cell signaling and cell motion. In Wuzhishan pigs (mini-type), the top ten biological processes were the cell cycle, RNA splicing, mRNA processing, regulation of the cell cycle, cell division, cell transport, small GTPase-mediated signal transduction, cell adhesion, phosphate transport and anti-apoptosis. These findings that indicated these breed pigs have significant difference in developmental trajectories and molecular changes. These difference maybe contribute to the phenotype variance of postnatal skeletal muscle between different breed pigs.

## Conclusion

In conclusion, this work is the first study to identify the involvement of miRNAs in the regulation of prenatal skeletal muscle development in different pig breeds. Differentially expressed mRNAs and miRNAs were identified, and their co-expression and interaction prediction were analyzed. The miRNAs and targets identified in this study are resources that can be used to understand the miRNA regulation of mammalian muscle development and phenotype variance between breeds. Our results are the first findings to indicate that miRNAs play an important role in prenatal skeletal muscle development and phenotypic differences in postnatal muscle between distinct types of pigs. The challenge for future studies will be to identify the relevant targets of miRNAs and to determine how miRNAs contribute to the regulation of skeletal muscle growth and phenotype variance.

## Materials and Methods

### Ethics statement

All animal procedures were performed according to protocols approved by The Hubei Province, P. R. China for Biological Studies Animal Care and Use Committee.

### Animal and RNA preparation

Landrace, Tongcheng and Wuzhishan gilts (at least nine sows for each breed) were mated with the boar of each corresponding breed. The sows were then sacrificed at a commercial slaughterhouse at 33, 65 and 90 dpc (three sows for each stage of each breed). All embryos were collected from sows at first parity. The longissimus muscle samples were immediately collected from fetuses. All samples were snap-frozen in liquid nitrogen and stored at –80°C until further analysis. Total RNA was isolated from frozen tissue samples using TRIzol (Invitrogen, Carlsbad, USA) according to the manufacturer’s instructions. Total RNA was treated with RNase-free DNase I to remove genomic DNA contamination. The RNA quality was evaluated by spectrophotometry and agarose gel electrophoresis. The same samples were used in all experiments.

### miRNA and mRNA oligonucleic array hybrid

The miRCURY^TM^ LNA miR Array (Exiqon, Vedbaek, Denmark) was used to carry out miRNA expression profiling. The experiment was performed using individual RNA samples (n = 3) isolated from different prenatal skeletal muscle specimens. Labeled targets obtained from 5 μg of total RNA were used for hybridization on each microarray. Purified RNA was labeled with a miRCURY^TM^ LNA miR Array Labeling kit (Exiqon, Vedbak, Denmark). The Hy3^TM^-labeled samples and Hy5^TM^-labeled reference pool RNA samples were then mixed pair-wise and hybridized to the miRCURY LNA array 10.0 (Exiqon, Vedbak, Denmark). All samples and replicates were analyzed on separate miRNA microarrays. The hybridization was performed according to the miRCURY LNA array manual. Following hybridization, the slides were washed using a wash buffer kit (Exiqon, Vedbak, Denmark), dried and scanned on a GenePix 4000B array scanner (Molecular Devices Co., Sunnyvake, CA, USA). GenePix pro V6.0 software was used to read the raw intensity of the image.

To analyze mRNAs expression, genome-wide mRNA expression profiles were obtained by pig oligoarray analysis ( http://www.pigoligoarray.org/) on the same samples used for miRNA profiling. The process was carried out according to the manufacturer’s instructions. Briefly, double-stranded complementary DNA (cDNA) was synthesized from total RNA. The hybridized probe array was subsequently stained and scanned by a Genechip Scanner 3000. We used the robust multi-array analysis expression measure that represents the log transform of (background corrected and normalized) intensities of GeneChips^73^. The median pixel intensities were background subtracted. Hybridization signals that failed to exceed the average background value by more than two standard deviations (Signal > Mean + 2 SD) were excluded from analysis. In the three duplicate slides, probe signal > Mean + 2 SD was classified as detected. Expression values were normalized using the Robust Multichip Average methodology^74^. To acquire the expression value, data were normalized between chips using the quantile normalization method. Statistical analysis was conducted using ANOVA for multiple comparisons. A *P*-value < 0.05 and a fold change of >1.5 were used as thresholds of significance for differential expression.

### qPCR experiments

Three differentially expressed miRNAs (miR-133b, miR-206 and miR-302b) were selected for data verification of the miRNA array. Expression of these mature miRNAs was assayed using stem-loop reverse transcription (RT) followed by qPCR analysis as previously described^75^. Gene-specific PCR forward primers and a universal PCR reverse primer were designed according to the miRNA sequences. The expression of the U6 small nuclear RNA gene was used as an internal control^76^. All primers used for stem-loop RT-PCR are shown in Table 5.

Twelve differentially expressed mRNAs (*IGF2*, *TPM3*, *COL15A1*, *CSRP3*, *LAMA2*, *LAMA3*, *LAMB2*, *UCHL1*, *FNDC1*, *FEZ2*, *FGL2* and *IGFBP7*) were used for validation of expression profiling. RT reactions were performed with total RNA (2 μg) using the ReverTra Ace kit (Toyobo, Osaka, Japan). The product was amplified in a reaction volume of 25 μl including 2 μl RT products, 1× reaction buffer, 1U ExTaq DNA polymerase (TakaRa, Japan) and 20 pmol of each primer. PCR reactions were performed for 20 cycles at 94°C for 45 s, 59°C for 30 s and 72°C for 30 s. The primer sequences are listed in Table 5. qPCR experiments were performed in triplicate for each sample as described above. The relative amounts of miRNA and mRNA were normalized against U6 snRNA and *GAPDH*, and the fold change for each miRNA and mRNA was calculated using the 2–ΔΔCt method^77^. The data are presented as fold changes in gene expression normalized against the internal control and relative to the L33 sample.

### Bioinformatic analysis

The Short Time-series Expression Miner (STEM) software^78^ was used to analyze the expression patterns of differentially expressed genes during skeletal muscle development. We determined the enrichment of clusters by comparing the distribution of observed groups with those expected in a random permutation. Gene co-expression analysis was performed with k-core algorithm^79^ to determine which genes may play pivotal roles during prenatal skeletal muscle development in pigs. The genes from the most significant up-regulated and down-regulated clusters were selected to construct the co-expression network in each pig breed.

To identify the potential target mRNAs of the miRNAs, we used a combined computational prediction and experimental method based on paired miRNA and mRNA profiling. Potential targets for differentially expressed miRNAs were predicted within the untranslated region (UTR) sequences of inversely correlated target transcripts using the PicTar, TargetScan and miRanda algorithms, which are associated with the Sanger miRbase^80^. Subsequently, a classical Pearson’s correlation test was performed to identify the negatively correlated pairs between a particular miRNA and potential target mRNA expression^15^. The significance of each correlation was assessed by assuming that the correlation under the null hypothesis of no correlation follows a distribution with n-1 degrees of freedom, where n is the number of measurements in the expression profile^15^,^81^.

To organize genes into hierarchical categories and identify the miR-gene potential regulatory network, GO enrichment analysis was performed to identify biological processes^82^. The significance of enrichment of a list of target genes with genes belonging to a GO group was scored using a weighting algorithm. A two-sided Fisher’s exact test and a chi-square x^2^ test were used to classify the GO category, and the FDR was calculated to correct the P-value. We chose only GOs that had a p-value of <0.001 and a FDR of <0.05. For each category, enrichment was scored using the weighting algorithm with a list of target genes belonging to a certain category. Then, the miRNA-mRNA interactions analysis during prenatal skeletal muscle development, depicting the critical miRNAs and their targets, was established for each breed based on STRING v9.1 ( http://string-db.org/)^83^. Potential interaction of miRNA-mRNA was visualized using Cytoscape V2.7 ( http://cytoscape.org/)^84^.

### Plasmid construction

The human mRNA sequences of *CNN3* (NM_001839.3), *RAP2C* (NM_021183.3) and *MAP1A* (NM_002373.5), retrieved from the GenBank database ( http://blast.ncbi.nlm.nih.gov/Blast/), were selected as entries to search for homologous sequences in the pig expression sequence tags (ESTs) database. The porcine ESTs sharing more than 85% sequence identity with human mRNA were selected and assembled using the Seqman program (DNAStar, Inc., Madison, WI, USA). The sequences of *SFRS3* (XM_003128366.3) and *SMARCD* (NM_001244613.1) were selected for primers (Table 6).

For the wild-type construct, the 3′ UTR sequences of target genes were amplified from pig genomic DNA and cloned downstream of the Renilla Luciferase ORF in the psiCHECK-2 vector (Promega, USA) using the NotI and XhoI restriction sites. For the mutated-type construct, the mutant 3′UTR sequences of target genes, which sharing a 7-bp deletion in the conserved binding site, were synthesized and inserted into the psiCHECK-2 vector. Porcine miR-206 and miR-133b sequences were synthesized by GenePharma Company in Shanghai.

### Cell culture

PIEC (Institute of Biochemistry and Cell Biology, Chinese Academy of Sciences, P. R. China) were cultured in Dulbecco’s modified Eagle medium (DMEM) with high glucose (Gibco) supplemented with 10% fetal bovine serum (Gibco, USA), 1% glutamine (Gibco, USA), and 1% penicillin/streptomycin (Gibco, USA) at 37 °C in an incubator supplemented with 5% CO_2_.

### Luciferase reporter assays

To detect the interactions between target genes and miRNAs, a dual luciferase reporter assay was performed. PIEC were seeded at 8 × 10^4^ cells per well in 24-well plates, and 24 hours after plating, the cells were transfected using Lipofectamine™ 2000 (Invitrogen, Carlsbad, CA, USA) according to the manufacturer’s instructions. In each well, 100 ng of wild-type or mutant 3′UTR plasmid RNA vector and 20 pmol of miRNA mimics or negative control were co-transfected. After 36 h, the cells were harvested by adding 100 μL passive lysis buffer, and Renilla and firefly luciferase activities in cell lysate were measured with the dual luciferase assay system (Promega) in a TD-20/20 luminometer (Turner Biosystems, Sunnyvale, CA). The Renilla luciferase signal was normalized to the firefly luciferase signal. This process was performed in triplicate for each target vector.

### Western blotting analysis

To validate the target *CNN3* of miRNA *miR-206*, western blotting was used to analyze interaction of miRNA-mRNA at the protein level. PIEC were seeded in 6-well plates, and 48 h after transfection, the cells were harvested for protein extraction. The protein concentration was measured with a BCA protein assay (Pierce Chemical, Rockford, USA) according to the manufacturer’s protocol, and 20 μg of each sample was subjected to sodium dodecyl sulfate polyacrylamide gel electrophoresis (SDS-PAGE) on an SDS-acrylamide gel. Separated proteins were transferred to polyvinylidene fluoride (PVDF) membranes (Millipore, Billerica, USA) and incubated with primary antibody (Calponin 3 (H-55): sc-28546; 1:1000 dilution; Santa Cruz Biotech., Santa Cruz, CA, USA) followed by incubation with a horseradish peroxidase (HRP)-conjugated secondary antibody (1:10000 dilution; Zymed, San Diego, CA, USA). The membrane was re-probed with a primary antibody against *GAPDH* (1:3000 dilutions; Santa Cruz Biotech., Santa Cruz, CA, USA) as a control. The assay was repeated to confirm the result.

## Additional Information

**How to cite this article**: Tang, Z. *et al.* Integrated analysis of miRNA and mRNA paired expression profiling of prenatal skeletal muscle development in three genotype pigs. *Sci. Rep.* **5**, 15544; doi: 10.1038/srep15544 (2015).

## Supplementary Material

Supplementary Information

Supplementary Table S1

Supplementary Table S2

Supplementary Table S3

Supplementary Table S4

Supplementary Table S5

Supplementary Table S6

## Figures and Tables

**Table 1:** qPCR validation of three differentially expressed miRs in microarray data.

	miR-133b		miR-206		miR-302b	
	miR array	qPCR	miR array	qPCR	miR array	qPCR
L33	1.0	1.0	1.0	1.0	1.0	1.0
L65	2.2	9.3	2.1	20.0	0.8	0.8
L90	5.7	16.8	1.9	27.7	0.7	0.6
T33	2.3	2.2	1.2	3.9	1.0	1.0
T65	3.5	10.1	1.9	35.4	0.6	0.7
T90	7.3	15.7	4.0	69.7	0.5	0.5
W33	21.1	159.7	1.8	15.3	0.4	0.5
W65	14.1	18.7	5.5	44.0	0.4	0.5
W90	5.1	15.3	3.9	35.9	0.8	0.8

Note: The qPCR row provides the ratio of the 2^−ΔΔCt^ of L33^46^. For the L33 sample, the fold change in gene expression relative to the L33 equals one, by definition. miR array row provides the expression abundance of each breeds/stage sample relative to L33 in the microarray data. L, Landrace; T, Tongcheng; W, Wuzhishan; 33, 65 and 90 refer to days post coitus.

**Table 2:** qPCR validation of 12 differentially expressed genes in oligoarray data.

Gene	Method	L33	L65	L90	T33	T65	T90	W33	W65	W90
*COL15A1*	Pig oligoarray	1.00	2.29	5.89	1.39	2.16	5.64	0.54	3.51	6.24
	qPCR	1.00	1.96	1.95	0.88	1.51	1.56	0.41	1.00	1.78
*TPM3*	Pig oligoarray	1.00	0.27	0.44	1.31	3.14	2.78	0.06	0.36	0.50
	qPCR	1.00	0.34	0.24	1.07	3.42	2.16	0.00	0.68	0.92
*IGF2*	Pig oligoarray	1.00	2.14	4.45	1.13	2.20	2.11	0.19	2.23	3.55
	qPCR	1.00	1.80	2.38	1.11	2.13	2.89	0.00	3.90	4.43
*CSRP3*	Pig oligoarray	1.00	3.04	4.41	1.26	2.79	3.93	0.99	3.31	3.83
	qPCR	1.00	1.38	1.48	0.64	0.71	0.98	0.96	1.41	1.89
*LAMA2*	Pig oligoarray	1.00	3.41	3.84	1.21	2.27	3.41	0.25	2.88	3.77
	qPCR	1.00	1.21	1.56	0.58	0.76	1.15	0.75	0.87	0.82
*LAMA3*	Pig oligoarray	1.00	5.54	10.71	0.52	4.63	13.22	0.64	5.79	9.12
	qPCR	1.00	18.74	23.41	1.16	15.61	66.07	1.84	11.88	15.74
*LAMB2*	Pig oligoarray	1.00	1.82	3.62	0.56	1.03	1.81	0.10	0.60	2.08
	qPCR	1.00	1.55	1.74	1.92	2.03	2.55	1.19	1.55	1.68
*UCHL1*	Pig oligoarray	1.00	0.09	0.06	0.96	0.13	0.11	0.42	0.10	0.07
	qPCR	1.00	0.15	0.03	1.32	0.06	0.05	2.06	0.05	0.03
*FNDC1*	Pig oligoarray	1.00	1.86	0.98	0.72	1.90	0.75	0.21	1.23	1.07
	qPCR	1.00	2.15	0.60	0.44	1.33	0.48	0.38	0.64	0.32
*FEZ2*	Pig oligoarray	1.00	3.04	5.01	0.46	2.96	3.67	0.66	4.34	5.04
	qPCR	1.00	3.17	3.90	1.62	2.53	3.09	1.88	3.49	4.31
*FGL2*	Pig oligoarray	1.00	15.85	19.52	2.40	6.34	26.36	6.18	32.29	39.04
	qPCR	1.00	16.46	20.36	1.72	17.33	42.52	1.09	21.93	37.07
*IGFBP7*	Pig oligoarray	1.00	2.12	4.61	1.24	2.04	3.69	0.37	1.52	3.47
	qPCR	1.00	1.20	1.28	1.1	1.06	2.20	1.14	1.20	2.38

Note: The qPCR row provides the ratio of the 2^−ΔΔCt^ of L33^46^. For the L33 sample, the fold change in gene expression relative to the L33 equals one, by definition. The microarray row provides the expression abundance of each breeds/stage sample relative to L33 in the oligoarray data. L, Landrace; T, Tongcheng; W, Wuzhishan; 33, 65 and 90 refer to days post coitus.

**Table 3:** The most significantly enriched GO term of clusters in each pig breed.

Cluster	GenesperCluster	Most significantly enriched GO term	*P*-value
Landrace			
15	206	GO:0030239: myofibril assembly	4.20E-11
12	122	GO:0006941: striated muscle contraction	8.13E-08
0	128	GO:0006259: DNA metabolic process	4.40E-10
11	174	GO:0003012: muscle system process	2.56E-07
13	104	GO:0001944: vasculature development	2.09E-05
3	86	GO:0051301: cell division	2.10E-08
2	118	GO:0007010: cytoskeleton organization	4.22E-05
Total	938		
Tongcheng			
15	131	GO:0006941: striated muscle contraction	8.13E-08
13	89	GO:0022904: respiratory electron transport chain	3.23E-08
12	86	GO:0048519: negative regulation of biological process	5.93E-05
3	61	GO:0051301: cell division	1.22E-07
8	82	GO:0045333: cellular respiration	1.51E-14
7	103	GO:0070252: actin-mediated cell contraction	5.82E-08
2	65	GO:0051258: protein polymerization	6.03E-06
Total	617		
Wuzhishan			
11	35	GO:0007165: signal transduction	0.012
4	193	GO:0006006: glucose metabolic process	6.43E-06
15	220	GO:0006112: energy reserve metabolic process	5.99E-05
14	169	GO:1902589: single-organism organelle organization	4.92E-07
1	158	GO:0006091: generation of precursor metabolites and energy	1.66E-08
12	49	GO:0061053: somite development	5.77E-04
Total	824		

**Table 4:**
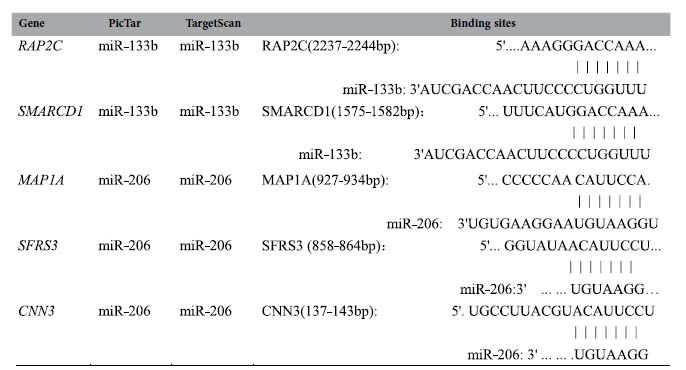
Predicted results on miRNA regulation of target genes.

**Table 5:** Primer sequences of miRNAs and genes selected for validation by qPCR.

Gene	Accession ID	Primer sequence	TM (°C)	Product size (bp)
miR-206	MI0013084	RT: CTCAACTGGTGTCGTGGAGTCGGCA		
		ATTCAGTTGAGTCACACA		
miR-133b	MI0013089	RT: CTCAACTGGTGTCGTGGAGTCGGCA		
		ATTCAGTTGAGTCGCTGGT		
miR-302b	MI0000772	RT: CTCAACTGGTGTCGTGGAGTCGGCAA		
		TTCAGTTGAGCTACTA		
miR-206	MI0013084	Forward 5′-GGGTGGAATGTAAGGAAG-3′	60	62
		Reverse 5′-CTCAACTGGTGTCGTGGAGTC-3′		
miR-133b	MI0013089	Forward 5′-GGGTTTGGTCCCCTTCA-3′	60	62
		Reverse 5′-CTCAACTGGTGTCGTGGAGTC-3′		
miR-302b	MI0000772	Forward 5′-GGGTAAGTGCTTCCATGTTT-3′	60	62
		Reverse 5′-CTCAACTGGTGTCGTGGAGTC-3′		
U6	EU520423	Forward 5′-GCTTCGGCAGCACATATACTAAAAT-3′	60	84
		Forward 5′-CGCTTCACGAATTTGCGTGTCAT-3′		
*GAPDH*	NM_001206359.1	Forward 5′-GGGCATGAACCATGAGAAGT-3′	60	233
		Reverse 5′-AAGCAGGGATGATGTTCTGG-3′		
*IGF2*	EST contig	Forward 5′-5′CACCATCCACCTCGTGACCT-3′	59	92
		Reverse 5′-GAGATGCCCGCAGACAGAA-3′		
*TPM3*	EST contig	Forward 5′-GGAACTCCAGGAAATCCAACTC-3′	59	214
		Reverse 5′-TTCTTCAGCAGCACTCAGACACT-3′		
*COL15A1*	EST contig	Forward 5′-CAGAAACCTGGTGACAGCATT-3′	60	236
		Reverse 5′-TTGAAATGGATGTCAGCGGAA-3′		
*CSRP3*	NM_001172368.1	Forward 5′-GATCGGCTATGGACAAGGTGC-3′	58	249
		Reverse 5′-CTCTTCCCACAGATGGCACAG-3′		
*LAMA2*	XM_001926517.2	Forward 5′-GCCCTGATTATGTGGGAGTTA-3′	60	220
		Reverse 5′-GCCTGTCCAGTCTGCCTTCGT-3′		
*LAMA3*	XM_003127861.1	Forward 5′-ACTTTGGAAGCACCTACTCAC-3′	60	186
		Reverse 5′-GACGACCATTTATCAAGGACAC-3′		
*LAMB2*	EST contig	Forward 5′-GAGGCAATGGTTGACACACA-3′	60	158
		Reverse 5′-AGGCTATTCCCTGCTCGTTT-3′		
*UCHL1*	NM_213763.2	Forward 5′-CAGTAGCCAATAATCAGGACA-3′	56	243
		Reverse 5′-TCCGACCATCAAGTTCATAGAG-3′		
*FNDC1*	EST contig	Forward 5′-ATCTGGCTGGAAAGAAACGCT-3′	60	230
		Reverse 5′-GTCCCAGTCCACAATGACGAAT-3′		
*FEZ2*	EST contig	Forward 5′-CAGGACCTTGCATTTGCTTAC-3′	62	235
		Reverse 5′-ATCTGACTGGGTGGGCGTTTC-3′		
*FGL2*	NM_001005152.2	Forward 5′-CCAACAATGAGACGGAGGA-3′	58	261
		Reverse 5′-CTGGGTCTCGGTTGTCGT-3′		
*IGFBP7*	NM_001163801.1	Forward 5′-CCATCGTGACACCCCCTAAG-3′	60	292
		Reverse 5′-GAAGCCTGTCCTTGGGAGTTA-3′		

**Table 6:** Information on primers corresponding to target genes and miRNA mimics.

Primer symbol	Primer sequence (5′-3′)	Size (bp)	TM (°C)
*CNN3-CDS(V)*-F	CTAGCTAGCATGACCCACTTCAACAAGGG	NheI	
*CNN3-CDS(V)-*R	CCGCTCGAGCTAATAATCAATGCCCTGGTCG	XhoI	
*CNN3-3*′*UTR*-F	GTTCACGGGGGAGCTCA	689	60.0
*CNN3-3*′*UTR*-R	GATACATTGGCACAAACAG		
*RAP2C-3*′*UTR*-F	AGATTGTAAGGGTGGAGGCA	532	57.0
*RAP2C-3*′*UTR*-R	AACAACTCCTAAACAGATGCCA		
*MAP1A-3*′*UTR*-F	CCCTCCGTATCTGAATGTCT	634	55.0
*MAP1A-3*′*UTR*-R	TCTTAGTCGGGCGGTAGTCT		
*SFRS3-3*′*UTR*-F	TTGTAGTTGAGCAAGCAGTC	721	54.0
*SFRS3-3*′*UTR*-R	ATGGACTTTTTGAACTGGCT		
*SMARCD1-3*′*UTR*-F	CCTGCCTTGGTCTTGCTT	1574	55.0
*SMARCD1-3*′*UTR*-R	ATTAAAACTGGGTGACATCG		
miR-133b	UUUGGUCCCCUUCAACCAGCUAU		
miR-206	UGGAAUGUAAGGAAGUGUGUGA		

Note: F, forward primer; R, reversed primers.
