# Supplementary material for: Integrated analysis of miRNA and mRNA paired expression profiling of prenatal skeletal muscle development in three genotype pigs
Source: Sci Rep. 2015 Oct 26;5:15544. doi: 10.1038/srep15544 (PMC4620456; doi:10.1038/srep15544)
Supplement: Supplementary Information [file srep15544-s1.doc]

**Supplementary Information for**

**Integrated analysis of miRNA and mRNA paired expression profiling of prenatal skeletal muscle development in three genotype pigs**

Zhonglin Tang^1,2^, Yalan Yang^1,2^,Zishuai Wang^1^, Shuanping Zhao^1,3^, Yulian Mu^1^, Kui Li^1,2^*

1. The State Key Laboratory for Animal Nutrition, Institute of Animal Science, Chinese Academy of Agricultural Sciences, Beijing 100193, China;
2. Agricultural Genome Institute at Shenzhen, Chinese Academy of Agricultural Sciences, Shenzhen, 518124, China;
3. Institute of Animal Science, Anhui Academy of Agricultural Sciences, Hefei, 230031, P. R. China.

Correspondence author: Kui Li*: The State Key Laboratory for Animal Nutrition, Institute of Animal Science, Chinese Academy of Agricultural Sciences, Beijing 100193, China. Email: [likui@caas.cn](mailto:likui@caas.cn)

**Supporting Information**

Table S1. The mRNAs differentially expressed during prenatal skeletal muscle development in Landrace pigs; L, Landrace pigs; 33, 65 and 90 refer to days post coitus.

Table S2. The mRNAs differentially expressed during prenatal skeletal muscle development in Tongcheng pigs; T, Tongcheng pigs; 33, 65 and 90 refer to days post coitus.

Table S3. The mRNAs differentially expressed during prenatal skeletal muscle development in Wuzhishan pigs; W, Wuzhishan pigs; 33, 65 and 90 refer to days post coitus.

Table S4. Differentially expressed miRNA and its target mRNAs during prenatal skeletal muscle development in Landrace pigs. L, Landrace pigs; 33, 65 and 90 refer to days post coitus.

Table S5. Differentially expressed miRNA and its target mRNAs during prenatal skeletal muscle development in Tongcheng pigs. T, Tongcheng pigs; 33, 65 and 90 refer to days post coitus.

Table S6. Differentially expressed miRNA and its target mRNAs during prenatal skeletal muscle development in Wuzhishan pigs. W, Wuzhishan pigs; 33, 65 and 90 refer to days post coitus.

Figure S1. The expression patterns of differentially expressed genes in Landrace pigs analyzed by STEM software. Each box represents a model expression cluster. The upper number in the cluster box is the model cluster number and the lower one is the p-value; seven expression patterns of genes showed significant p-values (P < 0.05) (colored boxes)

Figure S2. The expression patterns of differentially expressed genes in Tongcheng pigs analyzed by STEM software. Each box represents a model expression cluster. The upper number in the cluster box is the model cluster number and the lower one is the *p*-value; seven expression patterns of genes showed significant *p*-values (*P* < 0.05) (colored boxes)

Figure S3. The expression patterns of differentially expressed genes in Wuzhishan pigs analyzed by STEM software. Each box represents a model expression cluster. The upper number in the cluster box is the model cluster number and the lower one is the *p*-value; six expression patterns of genes showed significant *p*-values (*P* < 0.05) (colored boxes)


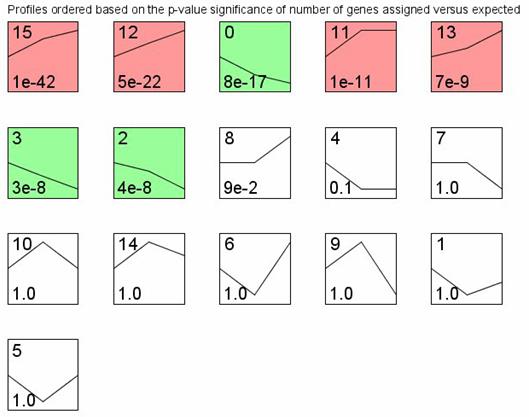


Figure S1. The expression patterns of differentially expressed genes in Landrace pigs analyzed by STEM software. Each box represents a model expression cluster. The upper number in the cluster box is the model cluster number and the lower one is the p-value; seven expression patterns of genes showed significant p-values (P < 0.05) (colored boxes)


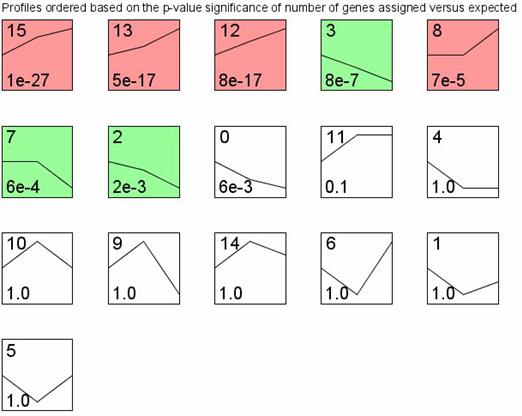


Figure S2. The expression patterns of differentially expressed genes in Tongcheng pigs analyzed by STEM software. Each box represents a model expression cluster. The upper number in the cluster box is the model cluster number and the lower one is the p-value; seven expression patterns of genes showed significant p-values (P < 0.05) (colored boxes)


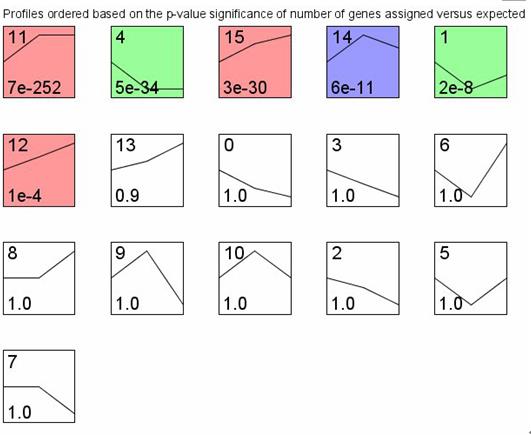


Figure S3. The expression patterns of differentially expressed genes in Wuzhishan pigs analyzed by STEM software. Each box represents a model expression cluster. The upper number in the cluster box is the model cluster number and the lower one is the p-value; six expression patterns of genes showed significant p-values (P < 0.05) (colored boxes)
